# Investigations Into the Metabolism and Elimination of Flmodafinil and Fladrafinil for Sports Drug Testing Purposes

**DOI:** 10.1002/dta.70100

**Published:** 2026-05-28

**Authors:** O. Krug, S. Guddat, C. Görgens, K. Walpurgis, F. Toma, A. Thomas, M. Thevis

**Affiliations:** ^1^ Center for Preventive Doping Research, Institute of Biochemistry German Sport University Cologne Cologne Germany; ^2^ European Monitoring Center for Emerging Doping Agents (EuMoCEDA) Cologne Germany

**Keywords:** blood, fladrafinil, flmodafinil, LC–MS, metabolism, urine

## Abstract

Flmodafinil (CRL‐40,940; 2‐[bis(4‐fluorophenyl)methylsulfinyl]acetamide) and fladrafinil (CRL‐40,941; 2‐[bis(4‐fluorophenyl)methylsulfinyl]‐N‐hydroxyacetamide) are structural analogs of modafinil. Their reported stimulating effects on the central nervous system have contributed to an increasing popularity outside the medical field, particularly for the enhancement of cognitive performance. In recent years, there has been a notable increase in interest within the sporting community regarding substances such as modafinil. Flmodafinil and fladrafinil are subjects of class “S6 stimulants” of the World Anti‐Doping Agency (WADA) Prohibited List, and therefore prohibited in‐competition. In this study, the elimination profiles of flmodafinil, fladrafinil, and their metabolites in urine and blood were investigated. Six volunteers ingested 20 mg of either flmodafinil or fladrafinil. Urine and blood (DBS) samples were analyzed by means of LC‐HRMS, where limits of detection between 0.2 and 4 ng/mL were accomplished. After ingestion of flmodafinil, the metabolites flmodafinil acid and flmodafinil sulfone were detected. Since fladrafinil acts as a prodrug for flmodafinil, these metabolites were likewise detected after ingestion of fladrafinil. It was found that urinary maximum concentrations ranged from 191 to 891 ng/mL for flmodafinil and from 52 to 111 ng/mL for fladrafinil. The maximum concentrations in blood ranged from 45 to 98 ng/mL for flmodafinil and from 11 to 35 ng/mL for fladrafinil. When fladrafinil is administered, a slight offset can be seen until the maximum concentration of flmodafinil is reached, related to the conversion of the prodrug fladrafinil to flmodafinil. In consideration of the relatively long detection windows of both the intact drugs and their metabolites, careful result interpretation is indicated in case of an AAF.

## Graphical abstract

This article investigates the metabolism and elimination of flmodafinil and fladrafinil, two fluorinated analogs of modafinil and adrafinil listed as S6 stimulants by the World Anti‐Doping Agency. After single‐dose administration to six volunteers, the compounds and their main metabolites were studied in urine and blood.

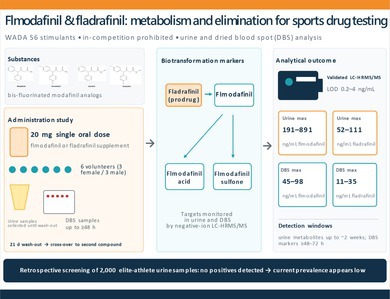

## 1 Introduction

**FIGURE 1:**
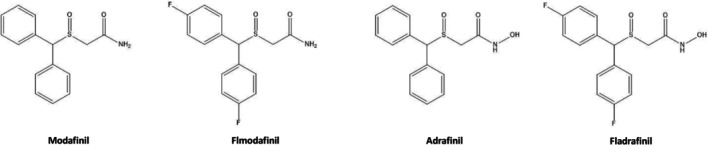
Chemical structures of modafinil, flmodafinil, adrafinil, and fladrafinil.

Stimulants are doping agents with wake‐promoting (“eugeroic”) effects and prohibited in‐competition [1]. They represent one of the first classes of performance‐enhancing drugs and constantly account for more than 10% of all adverse analytical findings (AAFs) reported by the World Anti‐Doping Agency (WADA) [2]. For instance, 495 of 3173 AAFs (= 16%) published in 2024 were related to stimulants such as methylphenidate (*n* = 70), cocaine (*n* = 64), and amphetamine (*n* = 56) [3]. The high sensitivity and selectivity of analytical methods and instruments allow for comprehensive screening procedures capable to reveal the misuse of stimulants as well as unintended doping due to the ingestion of contaminated/adulterated nutritional supplements [2]. Therefore, especially novel drugs such as flmodafinil (CRL‐40,940) and fladrafinil (CRL‐40,941) have an enormous potential for being misused in sports. Flmodafinil and fladrafinil are bisfluorinated analogs of modafinil and its N‐hydroxy derivative and prodrug adrafinil (Figure 1) [4]. They are supposed to be significantly more effective than the nonhalogenated analogs [5, 6], and were added to the 2026 edition of the WADA Prohibited List [7].

Because of the potential relevance of these compounds as performance‐enhancing agents in sports, the aim of this research project was to investigate the metabolism and elimination behavior of flmodafinil and fladrafinil, also in the light of the facile availability of supplements containing these compounds. Therefore, two administration studies with six healthy male and female volunteers were conducted, and dried blood spots (DBS) as well as urine samples were collected. Following extraction, all specimens were subjected to LC‐HRMS/MS analysis to study the elimination behavior of the drugs in urine and blood and to identify urinary metabolites of flmodafinil and fladrafinil. The obtained information should subsequently be employed to implement both drugs and/or their metabolites into existing doping control routine procedures [8, 9], which should be comprehensively characterized regarding method selectivity, sensitivity, robustness, etc. Moreover, approximately 2000 doping control routine samples were to be retrospectively evaluated for the presence of flmodafinil, fladrafinil, and their metabolites to determine the prevalence of these drugs in sports.

## 2 Material and Methods

### 2.1 Chemicals and Reagents

Reference material for flmodafinil and fladrafinil was purchased from Target Mol (Wellesley Hills, USA) and abcr (Karlsruhe, Germany). Flmodafinil and fladrafinil containing products were obtained from an internet‐based supplement provider. D_5_‐furosemide, methanol (MeOH), sodium hydroxide (NaOH), potassium permanganate (KMnO_4_), acetone, isopropanol, and hydrochloric acid (HCl) were purchased from Merck (Darmstadt, Germany). Acetonitrile (ACN) was obtained from VWR (Darmstadt, Germany) and formic acid (FA) from Carl Roth GmbH (Karlsruhe, Germany). *Tert*‐butyl methyl ether (*t*BME, distilled before use) was from KMF (St. Augustin, Germany). All chemicals and reagents used were of analytical grade. Deionized water (H_2_O) was produced with a Thermo Scientific Barnstead GenPure xCAD Plus system. Solid phase extraction (SPE) HR‐XCW‐ and Chromabond HLB‐cartridges were purchased from Macherey‐Nagel (Düren, Germany). Blood spot collection cards (QIAcard FTA DMPK‐C (100)) were obtained from Qiagen (Hilden, Germany), and Solofix Safety lancets were from B. Braun (Melsungen, Germany).

### 2.2 Metabolite Synthesis

The presumed main metabolites of flmodafinil, flmodafinil acid, and flmodafinil sulfone were synthesized in‐house according to established protocols [10]. For the synthesis of flmodafinil acid, 1 mg of flmodafinil was dissolved in 1.6 mL of 3‐M aqueous NaOH. The mixture was heated at 50°C for 30 min. Subsequently, the pH was adjusted to 1 with 780 μL of 6‐M HCl. Finally, flmodafinil acid was enriched by SPE (HLB cartridges), using MeOH/H_2_O (70:30; *v/v*) as eluent.

For the synthesis of flmodafinil sulfone, 1 mg of KMnO_4_ was added to a solution of 1 mg of flmodafinil solved in 1 mL of acetone. The mixture was incubated for 1 h at 55°C. After incubation, 50 μL of isopropanol was added to decompose the excess potassium permanganate. The solution was then diluted with 4 mL of water and extracted with 4 mL of *t*BME. Finally, the organic fraction was dried at 60°C under a stream of nitrogen and reconstituted in 1 mL of MeOH.

### 2.3 Product Testing

The quantification of flmodafinil and fladrafinil in nutritional supplements (labeled to contain 50 and 100 mg/mL, respectively) was performed by using a standard addition method. Aliquots (10 μL) of the liquid supplements were dissolved and diluted in ACN/H_2_O (50:50; *v/v*) by a factor of 2000 and a factor of 4000, respectively. These 1‐mL aliquots were then fortified with 0, 0.2, 0.6, and 1.0 μg of the solved reference material to generate calibration samples containing 0% to 400% of the estimated drug content. The resulting calibration curve was finally used to determine the drug content of the supplements used for the administration study described below. No other compounds were detected in the supplements.

### 2.4 Sample Collection

**FIGURE 2:**
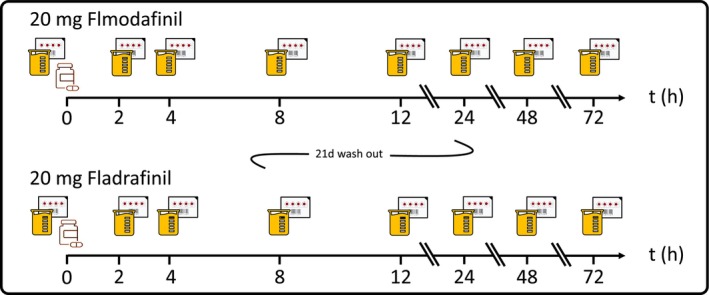
Scheme of flmodafinil/fladrafinil application and urine/DBS sample collection; fladrafinil application with the same volunteers after 3 weeks of washout. [Correction on 3 June 2026, after first online publication: Figure 2 has been replaced with the updated version to update the typo errors on “Flmodafnil” and “Fladrafnil”.]

After approval by the local ethics committee of the German Sport University Cologne (#089/2021), six volunteers were recruited (three male and three female), and an informed consent was obtained from all participants. Each administered a single dose of a nutritional supplement containing either 20 mg of flmodafinil or 20 mg of fladrafinil. DBS samples were collected before and up to at least 48 h following application as follows: 0 h—2 h—4 h—6 h—8 h—12 h—24 h—36 h—48 h. The fingertip was selected as the sampling site; the samples were collected using 20‐μL capillaries, applied to filter paper, and allowed to dry for at least 2 h. For analysis, the DBS were cut out in toto using scissors. Urine samples were collected until the intact compounds and main metabolites were no longer detectable. After the washout was confirmed (3 weeks), the volunteers administered 20 mg of the previously not ingested compound and followed the same sample collection protocol (Figure 2). Both urine and DBS samples were stored at −20°C until analysis.

### 2.5 Urine Sample Preparation

Chromabond HR‐XCW‐cartridges (45 μm and 1 mL) were conditioned with 1 mL of MeOH and 1 mL of H_2_O. To the urine samples (1 mL), 5 μL of a 1‐ppm D_5_‐furosemide solution was added as internal standard (ISTD), in accordance with established initial testing procedures routinely employed in the author's anti‐doping laboratory. After loading with urine, SPE cartridges were washed with 1 mL of H_2_O/FA (95:5; *v/v*). Subsequently, the analytes were eluted with 2 mL of MeOH/FA (95:5; *v/v*). Following evaporation to dryness, samples were reconstituted in 100 μL of H_2_O/ACN (95:5; *v/v*) for subsequent LC‐HRMS/MS analysis.

### 2.6 DBS Sample Preparation

Dried spots were punched out and extracted for 15 min with 1 mL of MeOH/H_2_O (60:40; *v/v*) using an ultrasonic bath. Here, also the ISTD (5 μL of a 1‐ppm D_5_‐furosemide solution) was added. Then, the solvent was evaporated at 50°C under a nitrogen stream and the resulting dry residue was reconstituted in 100 μL of H_2_O/ACN (95:5; *v/v*). Subsequently, the samples were centrifuged, and the supernatant was transferred into a vial for LC–MS analysis.

### 2.7 Liquid Chromatography‐Mass Spectrometry

LC‐HRMS/MS analyses of the target analytes were conducted using a Vanquish UHPLC chromatograph coupled via a heated electrospray ionization (HESI) source (300°C, −2.6 kV, in negative mode) to a Thermo Scientific Exploris 480 mass spectrometer. The chromatographic separation was achieved using an EC HPLC NUCLEOSHELL Biphenyl analytical column (100 × 2 mm; 2.7‐μm particle size; Macherey‐Nagel, Düren, Germany). The mobile phase was 0.1% FA (aq.) (solvent A) and 0.1% FA in ACN (solvent B). The gradient started at 100% A, decreased to 0% A over 5 min; the column was then flushed at 0% A for 2 min and subsequently re‐equilibrated at 100% A for 3 min. The total run time was 10 min. The flow rate was set to 0.3 mL/min; the injection volume was 5 μL. The ion source was operated with sheath, auxiliary, and sweep gas flows of 30, 10, and 0 units (arbitrary). The ion transfer tube was operated at 300°C, and the vaporizer temperature was 320°C.

The analysis was conducted using targeted MS/MS mode with a collision energy (CE) of 25 and a precursor isolation window of 1.3 *m/z*. Furthermore, full scan data were acquired at a resolution of 60,000 full width at half maximum (FWHM) at *m/z* 50–500. In Table 1, the monitored *m/z* values of the precursor and product ions are summarized.

**TABLE 1:** Target ions for LC–MS analysis.

Substance	MW [Da]	Precursor ion [M‐H]^−^ [*m/z*]	Product ion [*m/z*]	RT [min]
Flmodafinil	309.0635	308.0562	258.0736 58.0298	4.06
Fladrafinil	325.0584	324.0511	120.9838 74.0247	3.96
Flmodafinil acid (in‐house synthesis)	310.0475	309.0402	203.0682 105.9732	4.29
Flmodafinil sulfone (in‐house synthesis)	325.0584	324.0511	120.9841 79.9575	4.39

### 2.8 Method Validation

Both methods were validated according to current WADA guidelines [11], and the following parameters were determined:

#### 2.8.1 Selectivity

Selectivity was determined by analyzing 10 different blank urine/DBS samples (five male and five female samples) to probe for interfering peaks in the selected XICs at the expected retention times.

#### 2.8.2 Reliability

The reliability was investigated by analyzing seven urine/DBS samples fortified with 25 ng/mL of flmodafinil and fladrafinil, which corresponds to 50% of the WADA minimum required performance level (MRPL) defined for stimulants [12].

#### 2.8.3 Limit of Detection (LOD) and Limit of Identification (LOI)

The LOD and LOI were estimated by analyzing six batches of samples (*n* = 7) fortified with decreasing concentrations of flmodafinil and fladrafinil as follows: (1) Urine: flmodafinil 0.25, 0.5, 2.5, 5, 12.5, and 25 ng/mL; fladrafinil 0.025, 0.05, 0.5, 5, 12.5, and 25 ng/mL; (2) DBS: flmodafinil 0.5, 2.5, 5, 12.5, 25, and 50 ng/mL; fladrafinil: 0.25, 0.5, 2.5, 5, 12.5, and 25 ng/mL. Based on the resulting data, the LOD was defined as the lowest concentration at which the most intense product ions 258 (flmodafinil) and 121 (fladrafinil) were detected at a rate of 95%. By contrast, the two most intense product ions 258 and 58 for flmodafinil and 121 and 74 for fladrafinil were evaluated to estimate the LOI (detection rate: ≥ 95%). The detection rate was calculated by dividing the number of samples in which the analyte was detected by the total number of replicates per concentration (*n* = 7). The detection rate was then plotted against the concentration levels. The concentration at which the sigmoidal curve intersected the 95% detection rate was reported as the LOD/LOI.

#### 2.8.4 Matrix Effects

The ion suppression/enhancement effects were evaluated by comparing the areas of two fortified matrix extracts with those of spiked solvent at concentrations of 50 and 100 ng/mL for both analytes.

#### 2.8.5 Carryover

Carryover was assessed by measuring a blank specimen directly after a sample containing 200 ng/mL of the analytes.

#### 2.8.6 Linearity

In urine, the linearity of flmodafinil and fladrafinil was investigated at concentrations of 5–1300 and 1–1000 ng/mL, respectively. DBS were fortified with drugs at concentrations of 5–1000 and 1–1000 ng/mL, respectively. Relative peak areas (analyte/ISTD) were used to construct a calibration curve and determine linearity by regression analysis. Linearity was determined multiple times using a minimum of 6 calibration points.

#### 2.8.7 Recovery

To estimate the methods' recovery, six urine samples fortified with 250 ng/mL of flmodafinil and 100 ng/mL of fladrafinil were prepared as described above and compared to six blank sample extracts spiked with the same analyte concentrations prior to LC‐HRMS/MS analysis. For DBS, concentrations of 500 ng/mL (flmodafinil) and 250 ng/mL (fladrafinil) were investigated. The ISTD (D_5_‐furosemide) was added as described under Section 2.5.

#### 2.8.8 Precision

To assess the intraday and interday precision of the assay, 3 × 6 samples fortified with three different concentrations of flmodafinil and fladrafinil (urine: 10, 100, and 250 ng/mL of flmodafinil and 5, 20, and 200 ng/mL of fladrafinil; DBS: 10, 100, and 200 ng/mL of flmodafinil/fladrafinil) were analyzed on three consecutive days. Then, the precision was assessed by calculating the variation in absolute peak areas and reported as the coefficient of variation (CV).

#### 2.8.9 Stability

The stability of the analytes was assessed by repeatedly analyzing the working solutions stored at 4°C during a period of 3 months. Additionally, instrument stability was evaluated by reanalyzing the extracts of validation samples before and after a minimum of 14 days of storage at 10°C in the autosampler.

### 2.9 Prevalence in Elite Sports

In order to determine the prevalence of Flmodafinil and Fladrafinil misuse in elite sports, 2000 previously anonymized in‐competition doping control samples from elite athletes were retrospectively evaluated using Trace Finder software version 5.0 (Thermo Fisher). The retrospectively analyzed LC/MS data were collected following sample preparation using established routine protocols, which are very similar in principle to the preparation described here: purification or concentration of the urine sample via SPE and measurement by LC‐(HR)MS. To verify the suitability of the data and to determine m/z ranges for the ion chromatograms to be extracted, selected samples from the excretion study were analyzed using the routine protocols.

## 3 Results and Discussion

### 3.1 Flmodafinil Metabolite Synthesis

**FIGURE 3:**
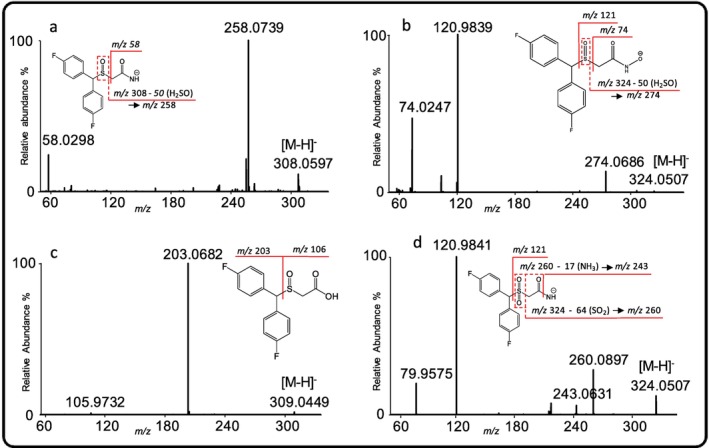
MS^2^‐mass spectra of (a) flmodafinil, (b) fladrafinil, (c) the metabolite flmodafinil acid, and (d) the metabolite flmodafinil sulfone, recorded at CE 25 in negative ionization mode.

The presumed main metabolites of flmodafinil and fladrafinil—flmodafinil acid and flmodafinil sulfone—were synthesized in‐house by using established protocols [10] and characterized by LC‐HRMS/MS. The corresponding product ion mass spectra are shown in Figure 3. Since neither the educts nor any synthesis by‐products were observed during LC‐HRMS analysis, a high yield and purity were assumed.

### 3.2 High Resolution/High Accuracy Mass Spectrometry of Flmodafinil, Fladrafinil, and Their Metabolites

The collision‐induced dissociation (CID) of flmodafinil, fladrafinil, and their metabolites was carried out at a CE of 25. The product ion mass spectra of flmodafinil ([M‐H]^−^: *m/z* 308) and fladrafinil ([M‐H]^−^: *m/z* 324) (Figure 3a,b) showed dissociation patterns attributable to the elimination of sulfanol (H_2_SO, −50 u), resulting in characteristic product ions at *m/z* 258 and 274, respectively. Additionally, the recorded product ion mass spectra showed ions at *m/z* 58 (flmodafinil), *m/z* 121 (fladrafinil), and 74 (fladrafinil), which are suggested to be related to the alkyl‐ (*m/z* 58 and 74) or alkylsulfonyl side chain (*m/z* 121).

In case of flmodafinil acid ([M‐H]^−^: *m/z* 309) (Figure 3c), the elimination of the aliphatic side chain was proposed to yield the product ion comprising the fluorinated biphenyl system at *m/z* 203. Also, a product ion plausibly assigned to the alkylsulfonyl side chain was observed at *m/z* 106. For flmodafinil sulfone ([M‐H]^−^: *m/z* 324) (Figure 3d), the elimination of sulfoxylic acid (H_2_SO_2_) was postulated, resulting in a product ion at *m/z* 260. Further, the elimination of ammonia (NH_3_) would consequently result in a product ion at *m/z* 243. Moreover, ions at *m/z* 121 corresponding to the alkylsulfonyl side chain and 80 corresponding to sulfur trioxide (SO_3_) were detected. Similar dissociation pathways were already described for the unfluorinated analogs by different research groups [12, 13] as well as aromatic sulfonamides [14].

### 3.3 Quantification of Flmodafinil and Fladrafinil in Nutritional Supplements

Prior to their usage for the administration studies, the nutritional supplements containing flmodafinil and fladrafinil were analyzed regarding the actual content of both drugs by using a standard addition approach. While the first product was labeled to contain flmodafinil at a concentration of 50 mg/mL, the quantification yielded a significantly higher amount of 95.7 mg/mL. For fladrafinil, the labeled content was 100 mg/mL, and the actual concentration was 93.1 mg/mL. Based on these results, the volumes required to administer 20‐mg doses of flmodafinil and fladrafinil were adjusted accordingly.

### 3.4 Method Validation

The validation results for both methods are summarized in Tables 2 and 3. The method for urine sample analysis was found to be highly selective, and no interfering signals were observed at the expected RT of flmodafinil and fladrafinil. For both analytes, the reliability was successfully demonstrated at a concentration of 25 ng/mL. Detection limits were estimated at 3.5 ng/mL for flmodafinil and 0.2 ng/mL for fladrafinil, and the corresponding LOIs were 7.5 and 22.7 ng/mL, respectively. Matrix effects for flmodafinil and fladrafinil were −44% and −54%. At a concentration of 200 ng/mL, no carryover was observed, and the method was found to be linear from 5% to 1300 ng/mL for flmodafinil (*R* ^2^ = 0.9882) and from 1 to 1000 ng/mL for fladrafinil (*R* ^2^ = 0.9935). Recovery rates were 52% for flmodafinil and 48% for fladrafinil. The intraday and interday precisions were investigated at flmodafinil concentrations of 10, 100, and 250 ng/mL and fladrafinil concentrations of 5, 20, and 200 ng/mL and yielded CVs below 10% for flmodafinil and below 22% for fladrafinil. Stability testing showed that both the standard solutions stored for 3 months at 4°C and the sample extracts kept in the autosampler for at least 14 days at 10°C are stable.

**TABLE 2:** Urine—results of method validation.

Validation parameter	Concentration(s) [ng/mL]	Flmodafinil	Fladrafinil
Selectivity	—	0/10	0/10
Reliability	25	7/7	7/7
LOD	0.25–25/0.025–25	3.5 ng/mL	0.2 ng/mL
LOI	0.25–25/0.025–25	7.5 ng/mL	22.7 ng/mL
Matrix effects	50 and 100	−44%	−54%
Carryover	200	—	—
Linearity	5–1300	*R* ^2^ = 0.9882	—
	1–1000		*R* ^2^ = 0.9935
Recovery	250	52%	
	100	—	48%
Intraday imprecision	5/10	3.6%–8.0%	8.7%–21.8%
	100/20	2.7%–3.9%	1.9%–4.3%
	250/200	1.8%–4.3%	1.7%–2.9%
Interday imprecision	5/10	9.3%	20.3%
	100/20	4.3%	4.1%
	250/200	5.9%	2.9%

**TABLE 3:** DBS—results of method validation.

Validation parameter	Concentration(s) [ng/mL]	Flmodafinil	Fladrafinil
Selectivity	—	0/10	0/10
Reliability	25	7/7	7/7
LOD	0.5–50/0.25–25	4 ng/mL	0.5 ng/mL
LOI	0.5–50/0.25–25	22 ng/mL	2 ng/mL
Matrix effects	50 and 100	−34%	−7%
Carryover	200	—	—
Linearity	5–1000	*R* ^2^ = 0.9845	—
	1–1000		*R* ^2^ = 0.9921
Recovery	500	34%	
	250	—	23%
Intraday imprecision	10	11.9%–20.0%	10.7%–18.1%
	100	4.4%–15.2%	6.0%–13.7%
	200	4.6%–9.0%	8.4%–11.4%
Interday imprecision	10	16.5%	14.6%
	100	10.9%	12.3%
	200	14.0%	13.7%

As shown in Table 3, the DBS method was also found to be highly selective and reliable at a concentration of 25 ng/mL. The LOD and LOI for flmodafinil were 4 and 22 ng/mL and for fladrafinil 0.5 and 2 ng/mL. Matrix effects were estimated as −34% for flmodafinil and −7% for fladrafinil. No carryover was observed at a concentration of 200 ng/mL, and the method was linear from 5–1000 and 1–1000 ng/mL for both analytes (flmodafinil: *R* ^2^ = 0.9845, fladrafinil: *R* ^2^ = 0.9921). With values of 34% (flmodafinil) and 23% (fladrafinil), recovery rates were significantly lower than in urine. For both drugs, the intraday and interday imprecisions were determined at concentrations of 10, 100, and 200 ng/mL, and the resulting CVs were equal to or below 20%. Again, sample extracts were found to be stable in the autosampler (10 C) for at least 14 days.

### 3.5 Administration Study

#### 3.5.1 Metabolism of Flmodafinil and Fladrafinil

**FIGURE 4:**
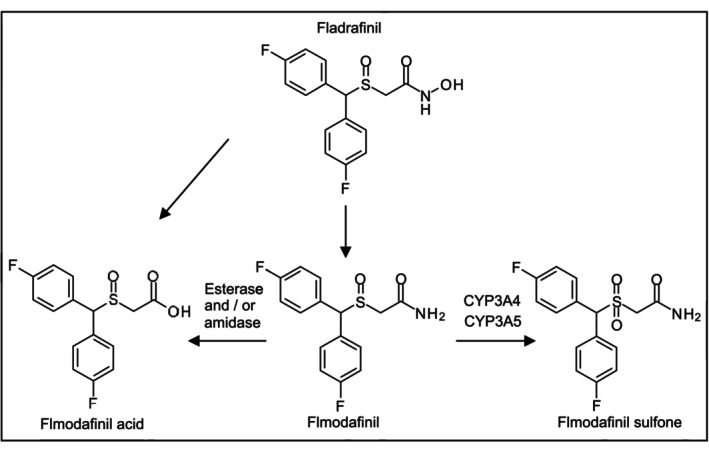
Metabolism of fladrafinil and flmodafinil.

Following administration of flmodafinil, the parent compound as well as flmodafinil acid and flmodafinil sulfone were detected in DBS and urine samples. These findings demonstrate that—in accordance with the literature published for its nonfluorinated analog [15, 16]—flmodafinil is metabolized to the inactive derivatives flmodafinil acid by esterases or amidases and flmodafinil sulfone by oxidation (Figure 4).

In the fladrafinil postadministration DBS and urine samples, flmodafinil, flmodafinil acid, and flmodafinil sulfone were identified in addition to the parent compound. Similar to adrafinil, fladrafinil is a prodrug metabolized to flmodafinil and flmodafinil acid [17]. The resulting flmodafinil is also converted to its main metabolites flmodafinil acid and flmodafinil sulfone.

#### 3.5.2 Elimination Data

**FIGURE 5:**
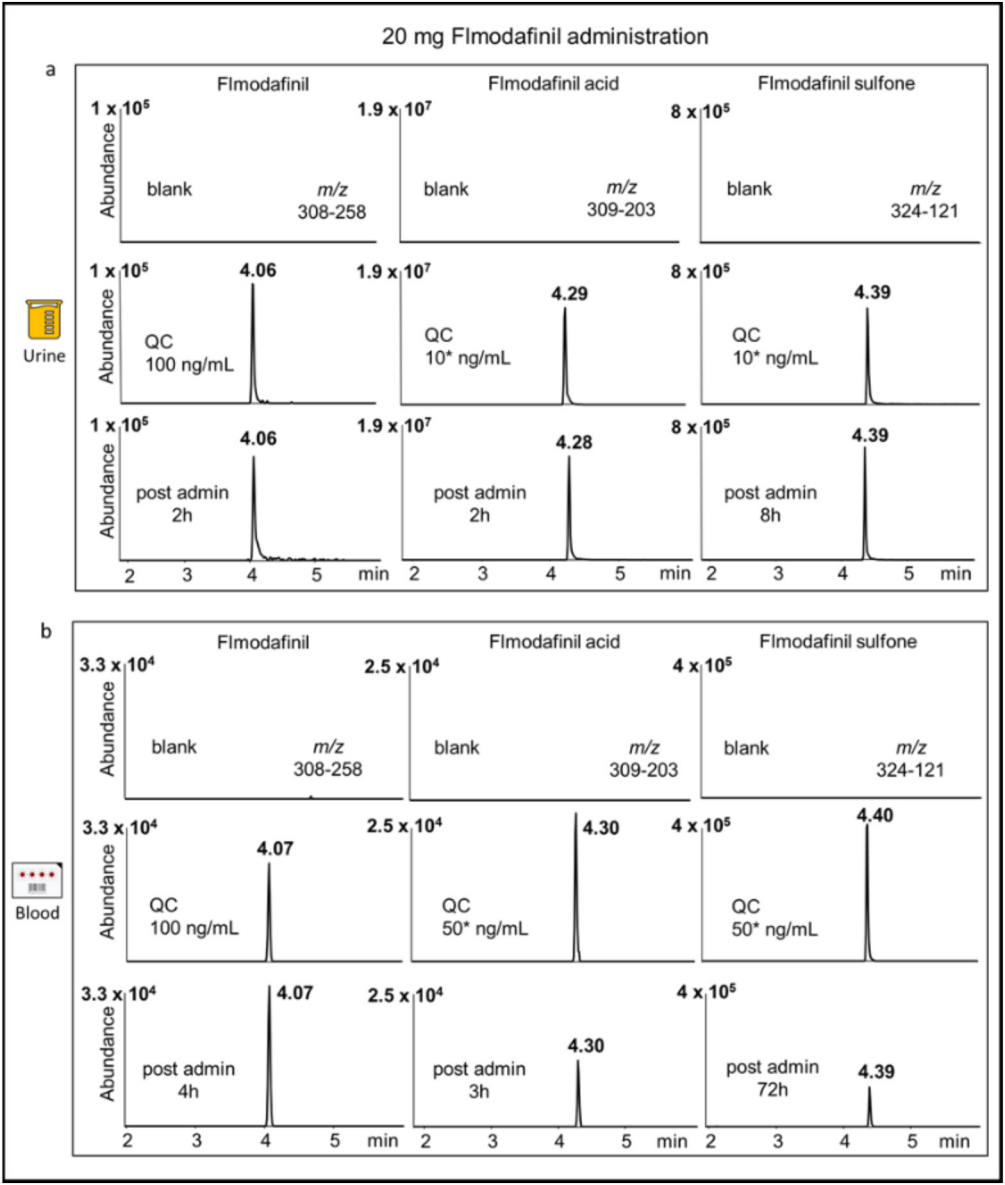
XICs of flmodafinil, fladrafinil, and their metabolites for blank, fortified, and postadministration urine (a) and DBS (b) specimens after a single dose of 20‐mg flmodafinil. *Concentration estimated as reference material was synthesized in‐house.

**FIGURE 6:**
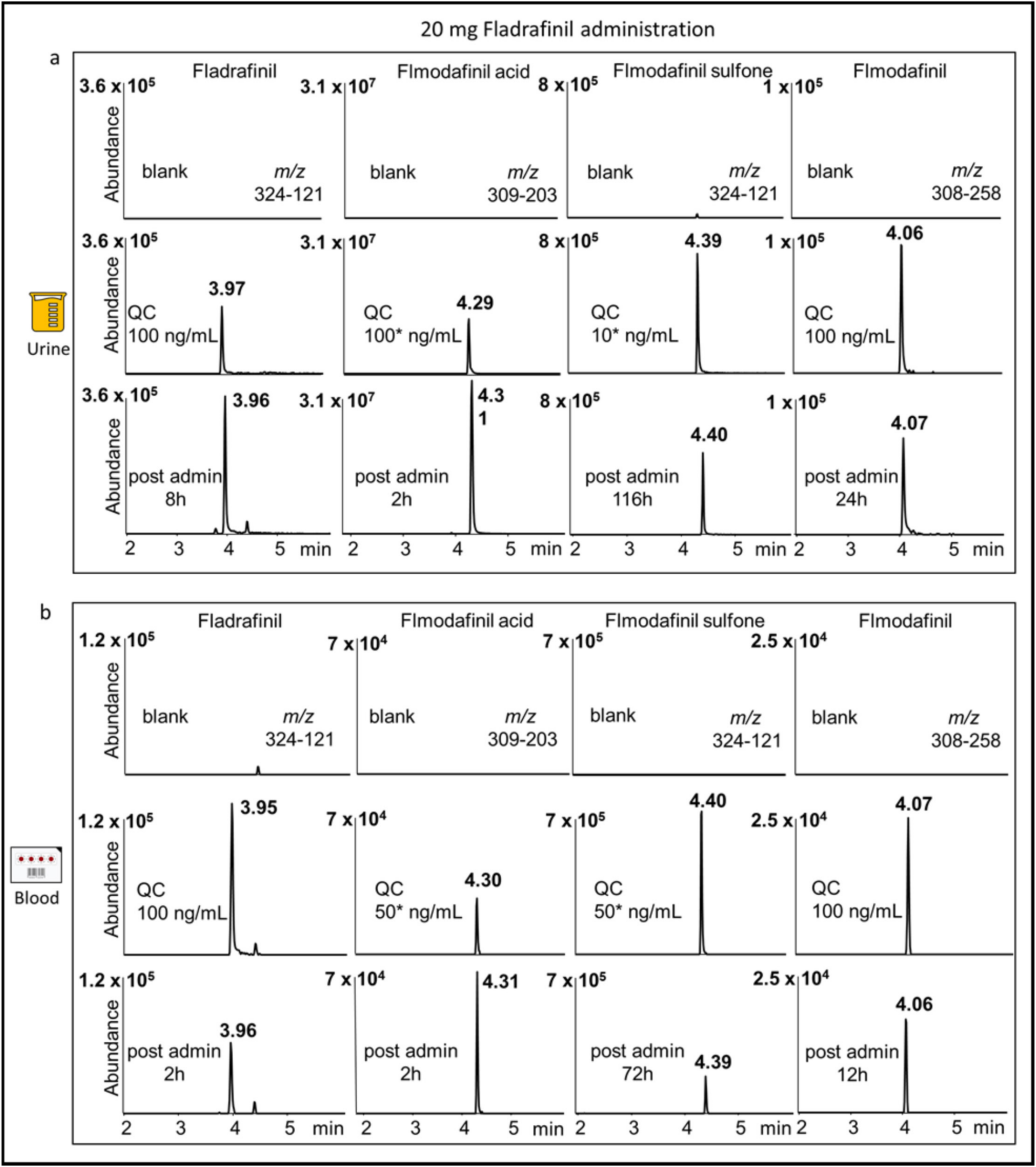
XICs of fladrafinil, flmodafinil, and their metabolites for blank, fortified, and postadministration urine (a) and DBS (b) specimens after a single dose of 20‐mg fladrafinil. *Concentration estimated as reference material was synthesized in‐house.

The postadministration urine and DBS samples collected according to the scheme shown in Figure 2 were extracted and analyzed as described above to quantify the amounts of flmodafinil and fladrafinil and qualitatively determine the main metabolites flmodafinil acid and flmodafinil sulfone. Figures 5 and 6 show exemplarily extracted ion chromatograms (XICs) of flmodafinil (*m/z* 308 → 258), fladrafinil (*m/z* 324 → 121), and their metabolites flmodafinil acid (*m/z* 309 → 203) and flmodafinil sulfone (*m/z* 324 → 121) for urine/DBS blank specimens, urine/DBS samples fortified with 10 to 100 ng/mL, and postadministration urine/DBS samples collected between 2 and 116 h after drug ingestion. The depicted excretion study samples were selected on the basis of the observed signal intensities in comparison to the QC samples. Under the described LC conditions, the active substances and their metabolites elute between 3.97 and 4.39 min. The concentrations of the metabolites were only estimated because no certified standard was commercially available, and reference material therefore had to be synthesized in‐house.

**FIGURE 7:**
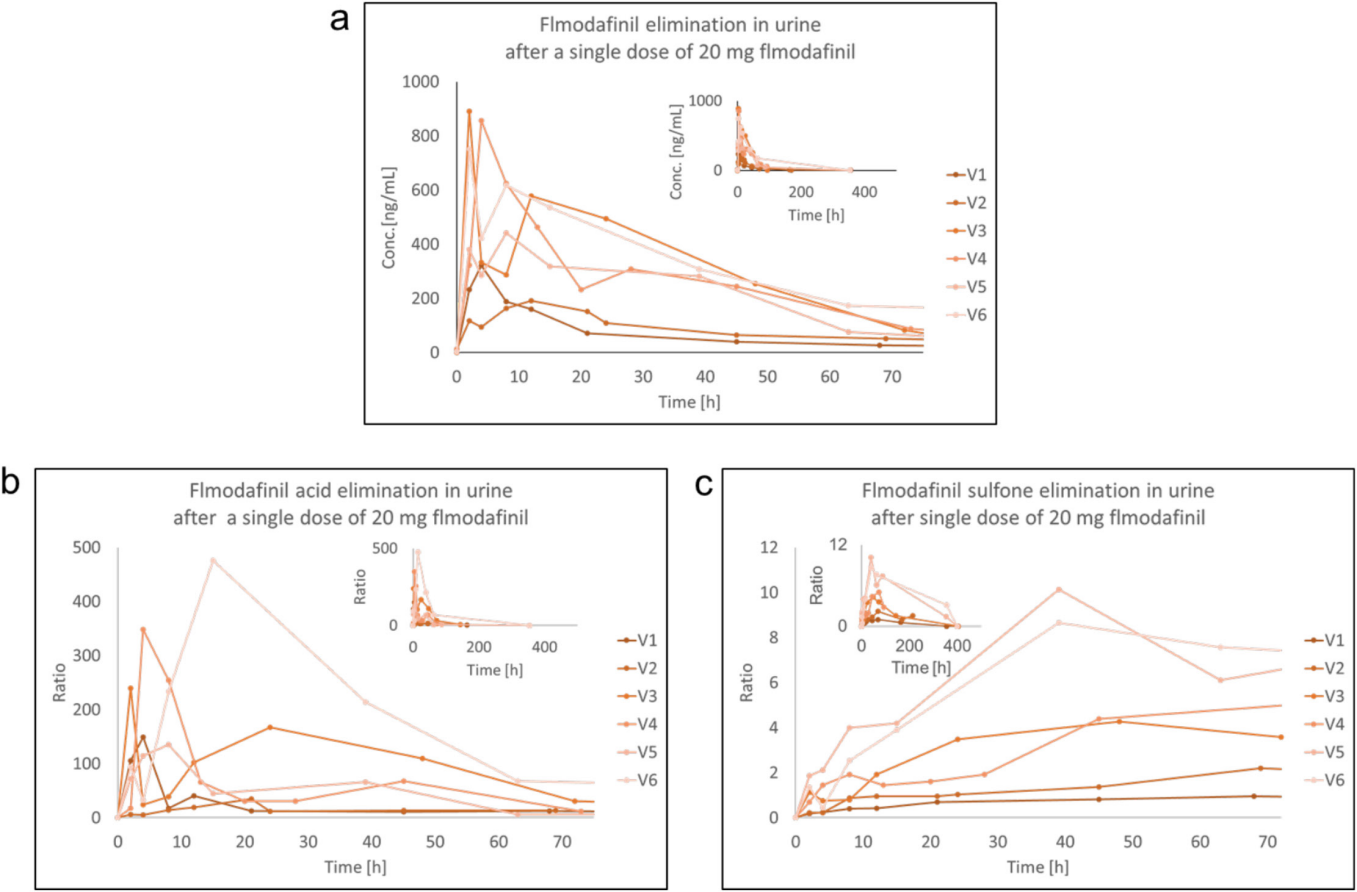
Elimination profiles of flmodafinil (a), flmodafinil acid (b), and flmodafinil sulfone (c) in urine following a single‐dose administration of 20 mg of flmodafinil. Insets show the complete time period of sample collection.

**FIGURE 8:**
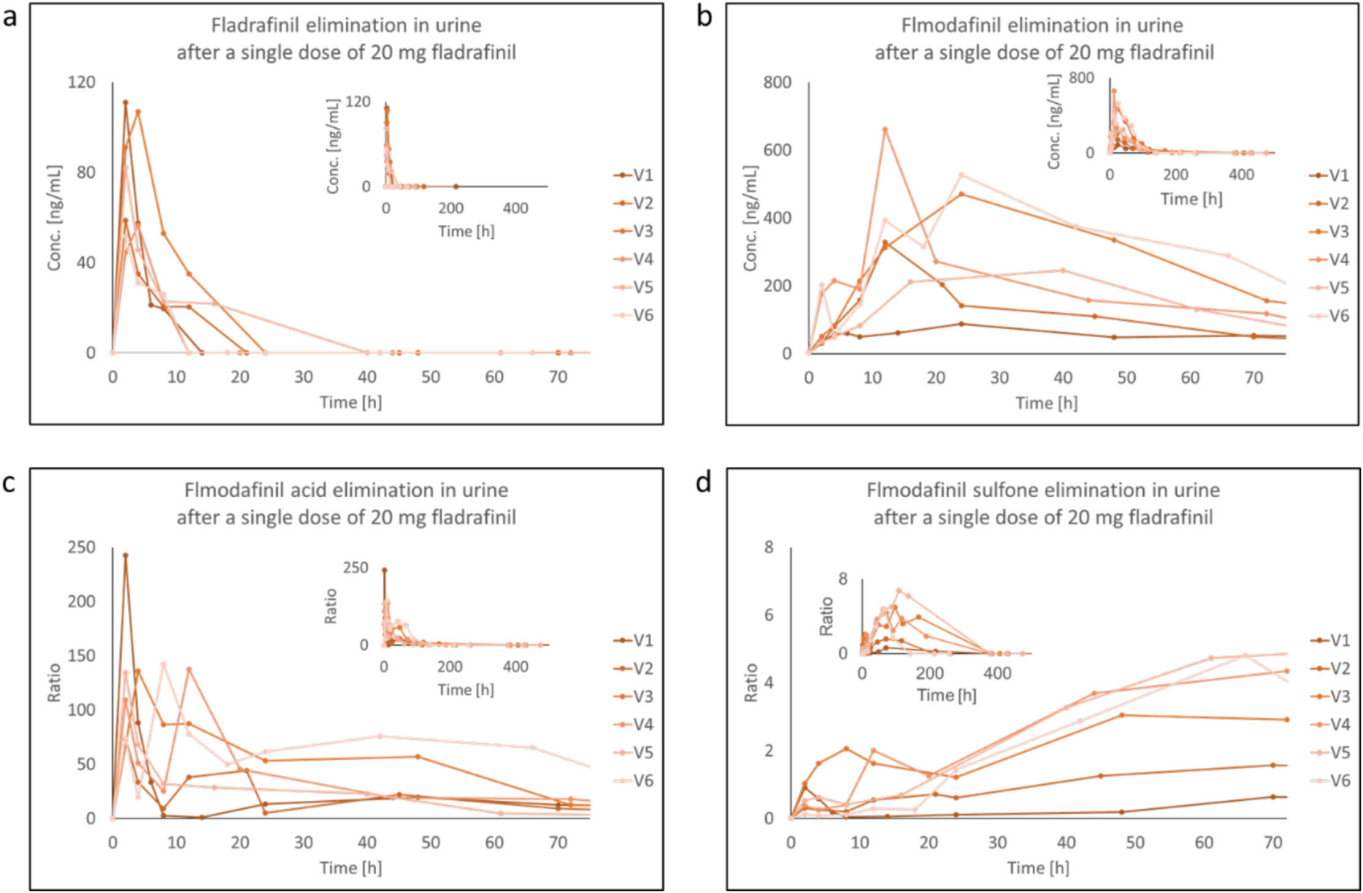
Elimination profiles of fladrafinil (a), flmodafinil (b), flmodafinil acid (c), and flmodafinil sulfone (d) in urine following a single‐dose administration of 20 mg of fladrafinil. Insets show the complete time period of sample collection.

**FIGURE 9:**
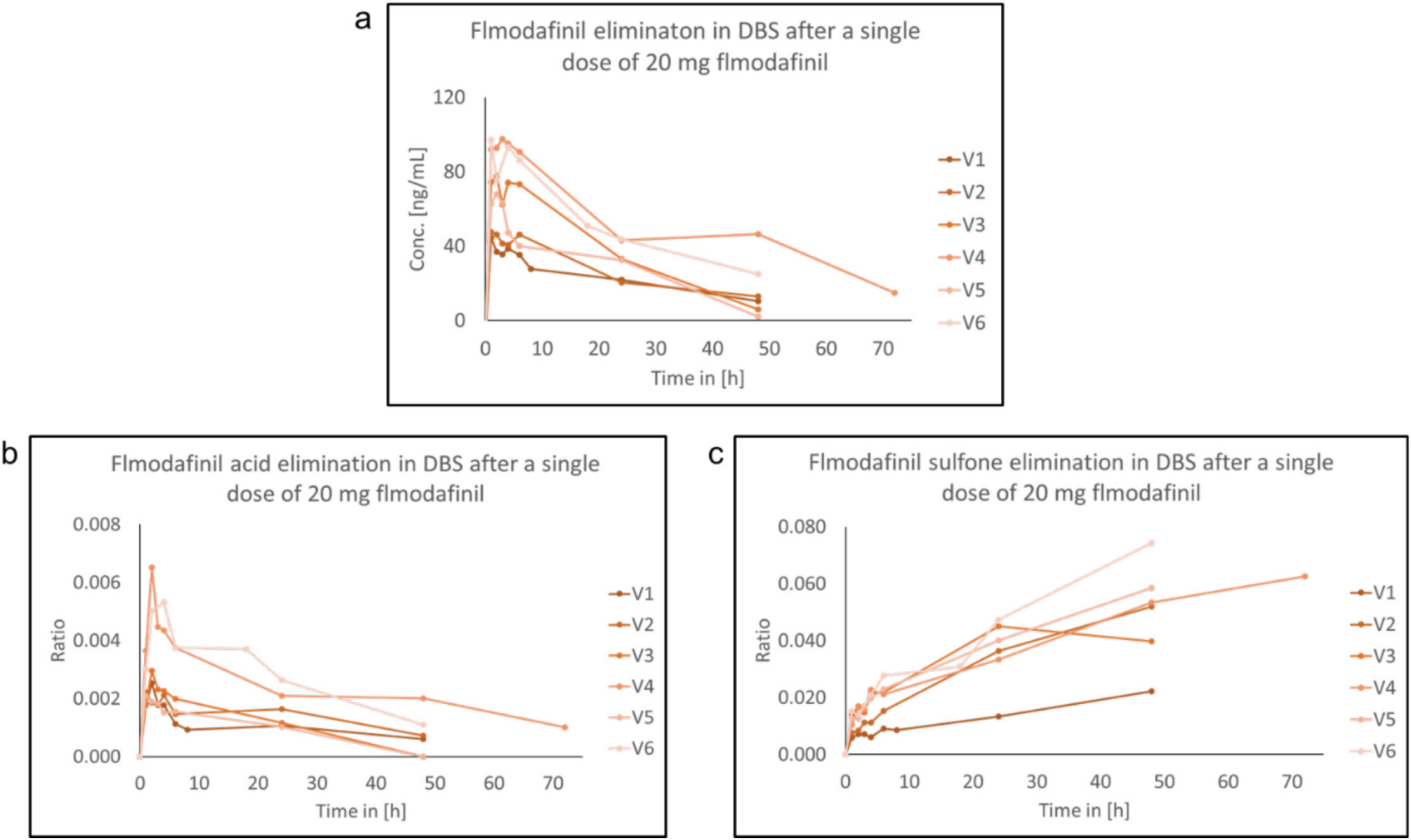
Elimination profiles of flmodafinil (a), flmodafinil acid (b), and flmodafinil sulfone (c) in DBS following a single‐dose administration of 20 mg of flmodafinil.

**FIGURE 10:**
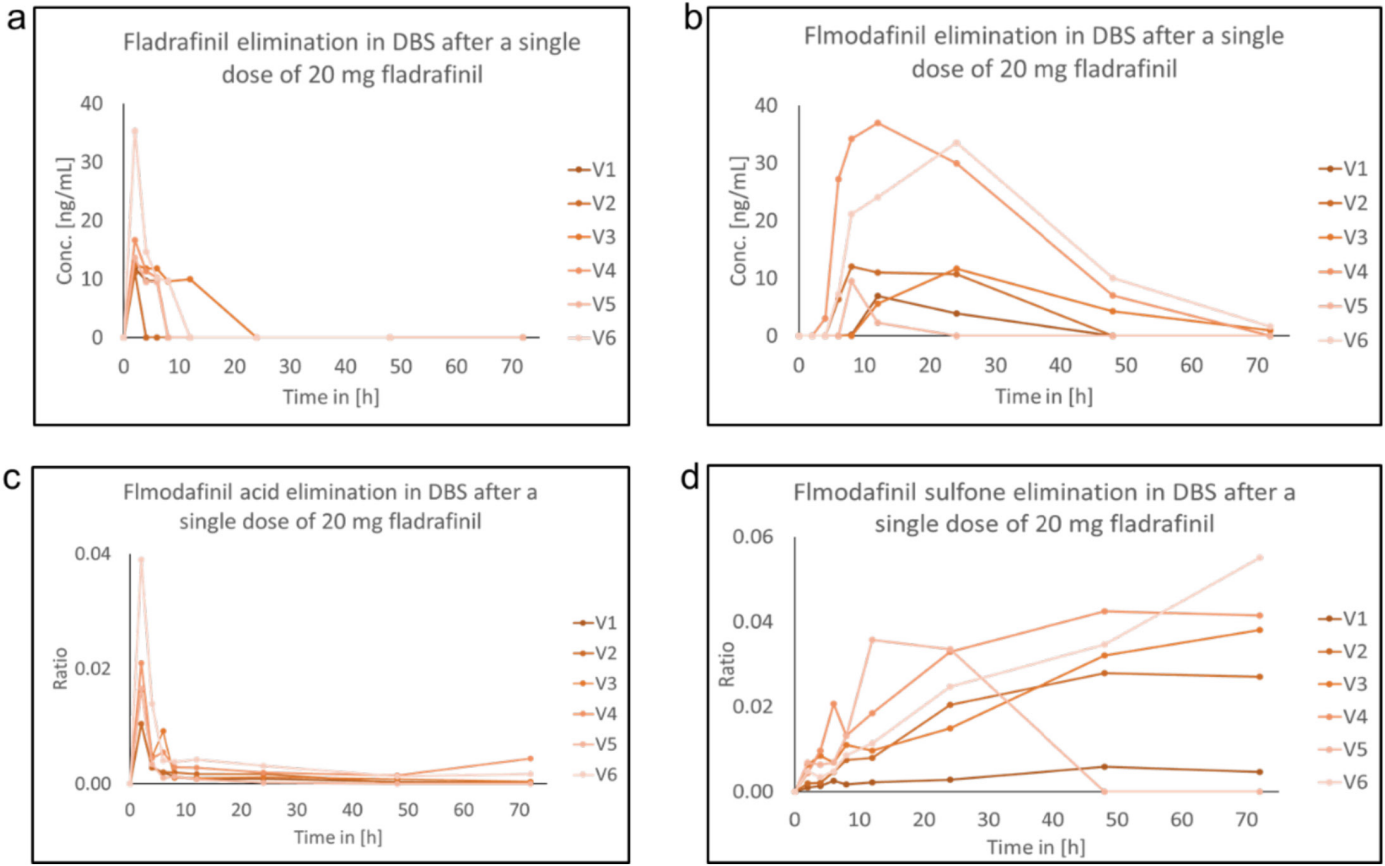
Elimination profiles of fladrafinil (a), flmodafinil (b), flmodafinil acid (c), and flmodafinil sulfone (d) in DBS following a single‐dose administration of 20 mg of fladrafinil.

The elimination profiles of the target analytes in urine and blood (DBS) are shown in Figures 7, 8, 9, 10. In the available urine samples, the maximum concentrations of flmodafinil ranged from 191 to 891 ng/mL and were observed within 2 and 12 h following drug application (Figure 7). The detection windows were up to 1 week for the parent compound and up to 2 weeks for the metabolites flmodafinil acid and flmodafinil sulfone. These findings are in accordance with an elimination study conducted in 2005, where three subjects received a modafinil single dose of 100 mg [18]. Maximum concentrations were 3.6–9.9 μg/mL and were observed between 2 and 8 h following oral administration. Despite the higher dose, detection windows were significantly shorter (48–72 h), which can be attributed to the significantly higher detection limit of 109 ng/mL.

By contrast, the maximum fladrafinil concentrations in the collected urine samples ranged from 52 to 111 ng/mL and were observed 2 or 4 h postadministration (Figure 8). While the parent compound was detectable in urine for up to 12 h, the metabolites flmodafinil, flmodafinil acid, and flmodafinil sulfone could be observed for up to 8 days (flmodafinil) and 2 weeks (flmodafinil acid and flmodafinil), respectively. Due to the conversion of the prodrug fladrafinil to flmodafinil, the peak concentrations of the latter were reached several hours later.

In the available DBS samples, maximum flmodafinil concentrations varied from 45 to 98 ng/mL and were observed after 1–3 h (Figure 9). Both the parent compound and the main metabolites were detectable for at least 48 h, and especially flmodafinil sulfone appeared to persist in the circulation significantly longer.

By contrast, the maximum concentrations of fladrafinil in the collected DBS specimens were 11 to 35 ng/mL and measured at time points 2 or 3 h (Figure 10). Due to the conversion to flmodafinil, its detection window was only 8–12 h, and all three metabolites could be detected significantly longer for at least 48 or 72 h. Noteworthy, no flmodafinil acid could be detected in the DBS specimens of one volunteer (V1).

### 3.6 Prevalence in Elite Sports

The LC‐HRMS/MS data from 2000 urine samples of an anonymized elite athlete testing pool (collection period ca. January 2023–December 2024) were retrospectively evaluated regarding the possible presence of flmodafinil, fladrafinil, and their metabolites by using Trace Finder software. As neither the parent compounds nor their main metabolites could be detected, the current prevalence appears to be low.

## 4 Conclusions

Flmodafinil and fladrafinil are derivatives of modafinil and can therefore be potentially misused as performance‐enhancing agents in sports. Therefore, the aim of this research project was to investigate the elimination behavior and metabolism of both drugs. For that purpose, both urine and DBS samples were collected within two in vivo administration studies with commercially available supplements containing either flmodafinil or fladrafinil. For the subsequent LC‐HRMS/MS analysis of the resulting samples, two extraction methods were developed and comprehensively characterized. In conclusion, regarding validation, the methods for determining the concentrations of analytes in urine and blood meet the requirements for use as a decision‐making aid in assessing stimulant abuse in doping cases. Besides the parent compounds, the metabolites flmodafinil acid and flmodafinil sulfone could be detected following drug ingestion, and valuable data on drug/metabolite concentrations and detection windows were generated. Even though the number of subjects is limited and statements regarding maximum concentrations or the dimension of the detection windows should therefore still be treated with caution, these can support the interpretation of AAFs, in particular, when scenarios of proven supplement contamination are discussed and supplement administration protocols exist.

In addition, reference material for the main metabolites flmodafinil acid and flmodafinil sulfone was synthesized in‐house. The next step will be to implement the parent compounds as well as the main metabolites into multianalyte screening procedures currently employed for doping control routine analysis.

## Funding

This project was conducted with the support of the World Anti‐Doping Agency (Montreal, Canada, grant #23C03MT), with the support of the Manfred‐Donike Institute for Doping Analysis (Cologne, Germany) and the Federal Ministry of the Interior and Community (Berlin, Germany).

## Conflicts of Interest

The authors declare no conflicts of interest.

## Data Availability

The data that support the findings of this study are available from the corresponding author upon reasonable request.
